# Lewis (y) Antigen Overexpression Increases the Expression of MMP-2 and MMP-9 and Invasion of Human Ovarian Cancer Cells

**DOI:** 10.3390/ijms11114441

**Published:** 2010-11-08

**Authors:** Limei Yan, Bei Lin, Lili Gao, Song Gao, Chuan Liu, Changzhi Wang, Yifei Wang, Shulan Zhang, Masao Iwamori

**Affiliations:** 1 Department of Obstetrics and Gynecology, Shengjing Hospital Affiliated to China Medical University, Shenyang 110004, China; E-Mails: leimei_yan@yahoo.com.cn (L.Y.); drgaolili@163.com (L.G.); song_gao@yeah.net (S.G.); drliuchuan@126.com (C.L.); shuluan_zhang@yeah.net (S.Z.); 2 Department of Obstetrics and Gynecology, the Second Affiliated Hospital of Dalian Medical University, Dalian 116027, China; E-Mails: changzhix_wang@yeah.net (C.W.); drwangyifei@163.com (Y.W); 3 Department of Biochemistry, Faculty of Science and Technology, Kinki University, 3-4-1 Kowakae, Higashiosaka, Osaka, 577-8502, Japan; E-Mail: iwamori@163.com

**Keywords:** Lewis (y) antigen, matrix metalloproteinases, tissue inhibitors of metalloproteinases, invasion

## Abstract

Lewis (y) antigen is a difucosylated oligosaccharide present on the plasma membrane, and its overexpression is frequently found in human cancers and has been shown to be associated with poor prognosis. Our previous studies have shown that Lewis (y) antigen plays a positive role in the process of invasion and metastasis of ovarian cancer cells. However, the mechanisms by which Lewis (y) antigen enhances the invasion and tumor metastasis are still unknown. In this study, we established a stable cell line constitutively expressing Lewis (y) antigen (RMG-1-hFUT) by transfecting the cDNA encoding part of the human α1,2-fucosyltransferase (α1,2-FUT) gene into the ovarian cancer cell line RMG-1, and investigated whether Lewis (y) antigen regulates the expression of matrix metalloproteinase-2 (MMP-2) and MMP-9, and tissue inhibitors of metalloproteinases (TIMP-1) and TIMP-2. We found that RMG-1-hFUT cells exhibited higher invasive capacities than their control cells. In addition, expression of TIMP-1 and TIMP-2 was down-regulated and expression of MMP-2 and MMP-9 was up-regulated. Anti-Lewis (y) antigen antibody treatment significantly reversed the expression of TIMP-1, TIMP-2, MMP-2 and MMP-9. Taken together, we provide the first evidence that down-regulation of TIMP-1 and TIMP-2 and up-regulation of MMP-2 and MMP-9 represents one of the mechanisms by which Lewis (y) antigen promotes cell invasion.

## 1. Introduction

Lewis (y) antigen is a difucosylated oligosaccharide containing two fucoses, and is carried by glycoconjugates (glycoproteins and glycolipids) on the plasma membrane. Its chemical structure is Fucα1 → 2Galβ1 → 4 [Fucα1 → 3]GlcNAcβ1 → R, which belongs to the A, B, H, Lewis blood group antigen family with specific fucosylation of the terminal end of carbohydrate structure catalyzed by the α1,2-fucosyltransferase (α1,2-FUT) [1,2]. Lewis (y) antigen is expressed predominately during embryogenesis. Under normal physiological conditions, its expression in adults is restricted to the surface of granulocytes and epithelium [3]. However, overexpression of Lewis (y) antigen is frequently found in human cancers and has been shown to be associated with poor prognosis [4,5]. Our previous studies using a stably α1,2-FUT stable transfected ovarian cancer cell line RMG-1-hFUT have shown that Lewis (y) antigen plays a positive role in the process of invasion and metastasis of ovarian cancer cells [6]. Nonetheless, the mechanisms by which Lewis (y) antigen enhances the invasion and tumor metastasis are still unknown.

Metastasis is a complex biological process consisting of a long series of sequential and interrelated steps, including the organized breakdown of the extracellular matrix (ECM) by matrix metalloproteinases (MMPs) [7,8]. MMPs belong to a rapidly growing family of structurally related endopeptidases capable of processing or degrading all ECM components. In particular, each ECM element is cleaved by a specific MMP or MMP group [9]. Among the human MMPs reported to date, MMP-2 and MMP-9 play vital roles in the degradation of the ECM because of their substrate specificity toward type IV collagen, the major component of basement membrane. High level expression of MMP-2 and MMP-9 has been frequently correlated with increased tumor invasion and poor prognosis in various types of human cancer [10,11]. The activity of MMPs is inhibited by specific tissue inhibitors of MMPs known as TIMPs. So far, four different TIMPs (TIMP-1, -2, -3 and -4) have been identified in humans [9]. Of these TIMPs, TIMP-1 and TIMP-2 are the best characterized TIMPs inhibitors of all MMPs [12]. In particular, TIMP-1 is more specific for MMP-9, and TIMP-2 regulates the activity of MMP-2 and MMP-9 in a concentration dependent fashion [13,14]. It is widely accepted that the degradation of ECM and, consequently, increased invasion capacity and metastatic potential of tumor cells results from the imbalance between the activities of these proteases and their inhibitors [15].

In this study, we established a stable cell line constitutively expressing Lewis (y) antigen (RMG-1-hFUT) by transfecting the cDNA encoding part of the human α1,2-fucosyltransferase (α1,2-FUT) gene into the ovarian cancer cell line RMG-1, and investigated whether Lewis (y) antigen regulates the expression of MMP-2, MMP-9, TIMP-1 and TIMP-2.

## 2. Results

### 2.1. Overexpression of Lewis (y) Antigen Enhances Migration of RMG-1 Ovarian Cancer Cells

**Figure 1.**
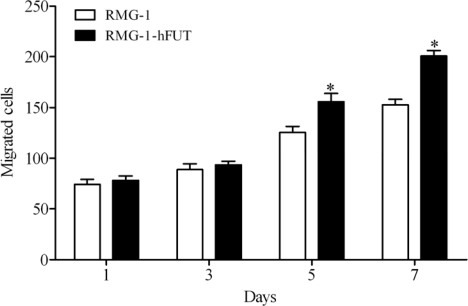
*In vitro* migration assay was performed by using 24-well transwell units coated with Matrigel. Invaded cell number was determined after cell seeding. * *P* < 0.05.

In our pervious study, RMG-1-hFUT cells established by transfection with the hFUT gene were shown to express Lewis (y) antigen at a significantly higher level than the original RMG-1 cells [16]. Thus, we next determined the impact of Lewis (y) antigen overexpression on the invasive ability of RMG-1 cells. *In vitro* transwell assay indicated that RMG-1-hFUT cells exhibited higher invasive ability than control cells (Figure 1). These data suggested that Lewis (y) antigen enhanced the metastatic potential of RMG-1 ovarian cancer cells.

### 2.2. Down-Regulation of TIMPs and up-Regulation of MMPs by Lewis (y) Antigen

**Figure 2.**
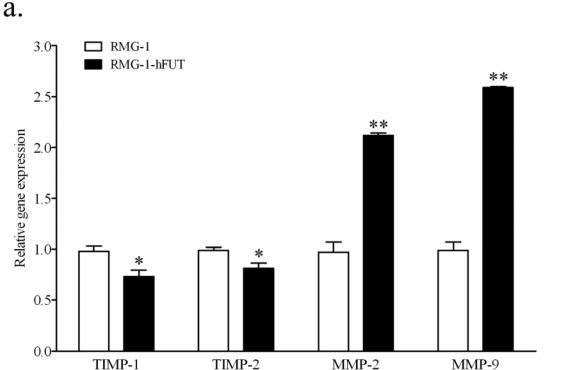

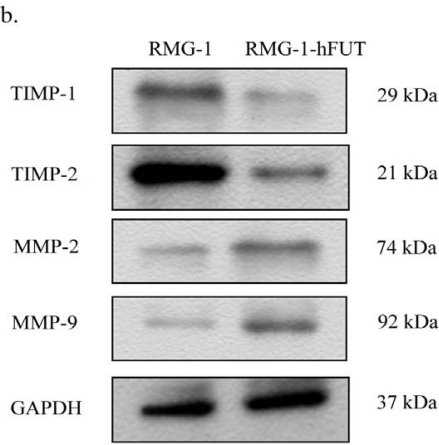
Expression of MMP-2, MMP-9, TIMP-1 and TIMP-2 in RMG-1 and RMG-1-hFUT cells shown by quantitative Real-Time RT-PCR (**a**) and Western blot analysis (**b**). (**a**) According to quantitative Real-Time RT-PCR, the mRNA level of these two TIMPs was significantly reduced, but the mRNA level of the two MMPs was markedly increased in RMG-1-hFUT cells, compared with RMG-1 cells (**b**) Protein levels of MMP-2, MMP-9, TIMP-1 and TIMP-2, determined by Western blot, were consistent with the mRNA levels. Representative blots are shown, and protein size is expressed in kDa. * *P* < 0.05, ** *P* < 0.01.

To explore the possible mechanisms of Lewis (y) antigen enhanced migration, we further tested the mRNA and protein levels of TIMP-1, TIMP-2, MMP-2 and MMP-9 in RMG-1-hFUT and RMG-1 cells by using quantitative Real-Time RT-PCR and Western blot analysis, respectively. As shown in Figure 2a, the mRNA level of these two TIMPs was significantly reduced, whereas the mRNA level of the two MMPs was markedly increased. Meanwhile, changes observed by Western blotting were in accordance with the findings in the quantitative real-time RT-PCR study (Figure 2b).

**Figure 3.**
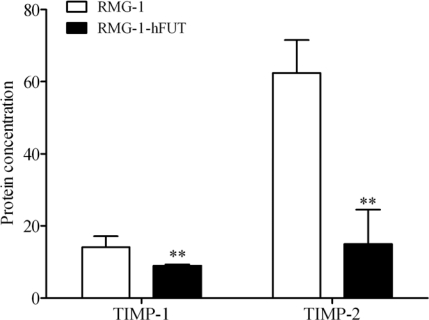
TIMP-1 and TIMP-2 concentrations as measured in the culture supernatants by ELISA. A diminution of TIMP-1 and TIMP-2 secretion was observed in RMG-1-hFUT when compared with RMG-1 cells. ** *P* < 0.01.

Furthermore, measurement of TIMP-1 and TIMP-2 concentrations in the culture supernatants by ELISA and statistical analysis of the data showed a diminution of TIMP-1 and TIMP-2 secretion in RMG-1-hFUT when compared with RMG-1 cells (Figure 3*, P* < 0.01).

**Figure 4.**
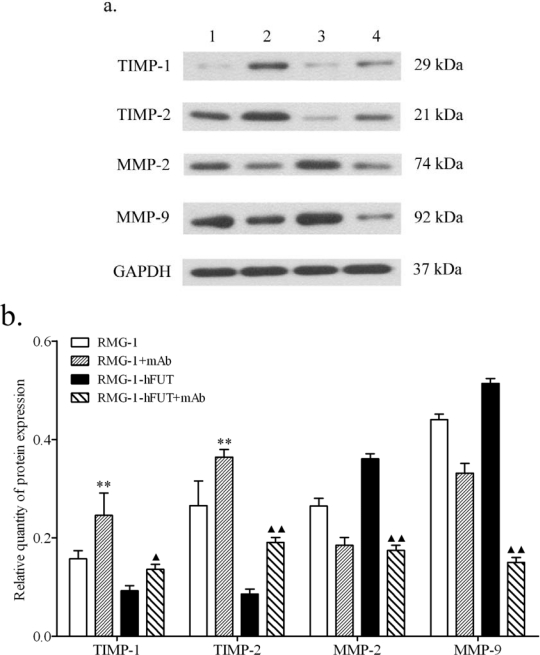
Protein levels of MMP-2, MMP-9, TIMP-1 and TIMP-2 in RMG-1 and RMG-1-hFUT cells treated with anti-Lewis (y) antibody. (**a**) A representative Western blot of three independent and reproducible experiments. Lane 1. RMG-1 cells; 2. RMG-1 cells + antibody; 3. RMG-1-hFUT cells; 4. RMG-1-hFUT cells + antibody. (**b**) Quantitative data were expressed as the intensity ratio target genes to GAPDH. * *vs.* RMG-1 cells, * *P* < 0.05, ** *P* < 0.01; ^▴^ *vs.* RGM-1-hFUT cells, ^▴^*P* < 0.05, ^▴▴^ *P* < 0.01.

To further demonstrate the association between Lewis (y) antigen and the expression of TIMP-1, TIMP-2, MMP-2 and MMP-9, anti-Lewis (y) antigen monoclonal antibody was used to block the Lewis (y) antigen present on the surface of RMG-1-hFUT cells. As shown in Figure 4, after RMG-1-hFUT cells were treated with anti-Lewis (y) antigen monoclonal antibody, the expression levels of TIMP-1 and TIMP-2 were increased, but the expression levels of MMP-2 and MMP-9 were decreased.

All these findings suggested that overexpression of Lewis (y) antigen inhibited the expression TIMP-1 and TIMP-2, but increased the expression of MMP-2 and MMP-9.

## 3. Discussion

As described in our previous papers, we successfully transfected the α1,2-FUT gene into human ovarian carcinoma-derived RMG-1 cells, which contain a significantly high amount of Lewis (x), the precursor of Lewis (y), and established RMG-1-hFUT cells with higher expression level of Le (y) compared with RMG-1 cells. Our further experiments demonstrated that RMG-1-hFUT cells not only exhibited increased proliferation and invasion capacity, but also showed high tolerance to common chemotherapy drugs for ovarian cancer, such as carboplatin, 5-fluorouracil and taxol [16–19]. However, the molecular mechanisms by which Lewis (y) causes these malignant properties of human ovarian cancer cells have not been completely understood. The present study is the first to address the mechanism by which Lewis (y) promotes tumor invasion and metastasis. We found that changes in expression of TIMP-1, TIMP-2, MMP-2 and MMP-9 are involved in the enhancement of cell invasion by Lewis (y).

Despite overexpression and invasion promoting ability of Lewis (y), and MMP-2 and MMP-9 being separately reported in various types of human cancer, a direct association between Lewis (y) and these two TIMPs has never been described. In this study, we showed that overexpression of Lewis (y) increased MMP-2 and MMP-9 expression to promote migration and invasion of ovarian cancer cells. Furthermore, we also found that the expression of TIMP-1 and TIMP-2 was inhibited by overexpression of Lewis (y). These results suggest that MMPs/TIMPs play a crucial role in Lewis (y) antigen-mediated cell invasion. Our previous microarray study indicated that a number of genes associated with metastasis are differentially expressed in RMG-1-hFUT cells, compared with those in RMG-1 cells [6]. In addition to MMPs and TIMPs, Lewis (y) antigen may also promote cell invasion via other mechanisms. Moreover, we also noted a significant increase of TIMP-1 and TIMP-2 in RMG-1 cell treated with anti-Lewis monoclonal antibody, which is probably because of the endogenous expression of Lewis (y) in RMG-1 cells [18].

More recently, Javier *et al*. have demonstrated that MMP-9, more specifically the hemopexin domain, has anti-apoptotic effects on cancer cells [20]. Thus, the elevation of MMP-9 not only contributes to the enhancement of cell invasion, but also maintains the malignant properties of RMG-1-hFUT cells. In addition, there is growing evidence that the epidermal growth factor receptor pathway and PI3K/Akt signal transduction pathway are key regulators of TIMP/MMP balance [21,22]. We have previously showed that Lewis(y) antigen stimulates the growth of ovarian cancer cells via regulation of the epidermal growth factor receptor pathway and the PI3K/Akt signaling pathway [18,19]. It therefore seems reasonable to propose that activation of the PI3K/Akt signaling pathway may represent one underlying mechanism for the Lewis (y) antigen-mediated cell invasion by regulating the expression of TIMPs and MMPs. However, the mechanisms by which Lewis (y) regulates the TIMPs and MMPs need further to be elucidated.

## 4. Experimental Section

### 4.1. Cell Culture

The human ovarian cancer cell line, RMG-1, was kindly provided by Professor Iwamori Masao (Tokyo University, Japan). RMG-1-hFUT cell line, highly expressing Lewis (y) antigen, was established by transfecting the pcDNA3.1 (-)-HFUT-H expression vector (containing a1,2-FUT gene) into RMG-1 cells as previously described [16,17]. Cells were maintained in Dulbecco’s modified Eagle’s medium (DMEM, Gibco, Grand Island, NY, USA) supplemented with 10% fetal bovine serum (FBS, Hyclone, Logan, UT, USA) at 37 °C in a humidified 5% CO_2_ atmosphere. Cells were routinely passaged, and cells at logarithmic growth phase were used for further experiments.

### 4.2. RNA Isolation and Quantitative Real-Time RT-PCR

Total RNA from treated cells was isolated using Trizol reagent (Life Technologies, Inc., Rockville, MD). RNA (2 μg) was converted to complementary DNA (cDNA) using the RT-PCR kit (TAKARA Bio., Dalian, China), according to the manufacturer’s protocol. Then the cDNA was subjected to Real-Time PCR analysis using the SYBR Green PCR Master Mix (TAKARA Bio., Dalian, China) on the ABI Prism 7500 Sequence Detection System (Applied Biosystems, Foster City, CA, USA). The PCR primer sequences were designed according to the human MMP-2, MMP-9, TIMP-1 and TIMP-2 gene sequences reported in GenBank and chemically synthesized (Table 1). The specificity of the PCR was confirmed by examining the dissociation reaction plot subsequent to Real-Time RT-PCR. Human glyceraldehyde-3-phosphate dehydrogenase (GAPDH) served as the constitutive control. PCR reactions of each sample were done in triplicate. Data were analyzed through the comparative threshold cycle (C_T_) method.

### 4.3. Western Blot Analysis

Total proteins from cells were separated on SDS-polyacrylamide gels and then electro-transferred to PVDF membranes. MMP and TIMP proteins were visualized by immunodetection using monoclonal MMP-1, MMP-2 (Abcam, Cambridge, MA, USA, dilution 1:2,000), TIMP-1, or TIMP-2 (Santa Cruz Biotechnology, CA, USA, dilution 1:2,000) antibodies. After subsequent incubation with horseradish peroxidase-linked secondary antibodies, the labeled proteins were detected using an enhanced chemiluminescence (ECL) kit (Amersham-Pharmacia, Freiburg, Germany).

### 4.4. Cell Migration Assay

Migration assays were conducted in 24-well plate applying Transwell cell culture chambers (8 mm pore size; Costar, Cambridge, MA, USA). Matrigel-coated Transwell inserts were prepared by adding 100 μL of Matrigel (250 μg/mL) to the Transwell and allowing the Matrigel to dry at 37 °C for 12 h. Harvested cells at a density of 1 × 10^5^ were seeded in the upper well with 100 μL medium supplemented with 10% FBS. The same medium was placed in the lower chamber. At the end of the 24-hour incubation at 37 °C, 5% CO_2_, cells on the top of the membrane were removed by swiping with a damp cotton swab, and cells that had migrated to the lower surface were fixed in methanol for 15 minutes at room temperature and stained with H&E. Cell migration was quantified by counting the migrated cells on the lower surface of the membrane in ten individual fields using a 400× objective.

### 4.5. Anti-Lewis (y) Antigen Antibody Blocking Test

Cultured RMG-1 or RMG-1-hFUT cells in exponential growth phase were harvested to prepare single cell suspension, and mouse anti-human Lewis (y) monoclonal antibodies (Abcam, UK) was then added at a final concentration of 10 μg/mL. After incubation at 37 °C for 30 min, cells were harvested. Proteins were isolated and Western blot analysis was performed to detect the expression of MMP-2, MMP-9, TIMP-1 and TIMP-2 proteins.

### 4.6. ELISA

TIMP-1 and TIMP-2 levels in the culture supernatants were assayed using commercially available human TIMP ELISA test kits (NeoBioscience, Shenzhen, China) according to the manufacturer’s recommendations. The results were finally expressed as the mean ng/mL ± standard errors.

### 4.7. Statistical Analysis

All experiments were performed in triplicate and all data are expressed as mean ± standard errors. Raw data were analyzed by the unpaired Student’s *t* test using SPSS 11.0 software (SPSS Inc., Chicago, IL, USA). A *P*-value < 0.05 was considered to be statistically significant.

## 5. Conclusions

In summary, we showed that overexpression of Lewis (y) promotes the invasion and metastasis of ovarian cancer cells, and down-regulation of TIMPs and up-regulation of MMPs may be involved in these processes. Although the specific mechanisms still need to be further studied, our results raise the possibility that inhibition of Lewis (y) antigen may suppress tumor invasion and metastasis.

## Figures and Tables

**Table 1.** Primer sequences used for quantitative Real-Time RT-PCR.

**Gene symbol**	**Sequences**	**Product size (bp)**
TIMP-1	F: 5’-GTTGTTGCTGTGGCTGATAG-3’	266
	R: 5’-TGTGGGACCTGTGGAAGTA-3’	
TIMP-2	F: 5’-CGCTCAAATACCTTCACAA-3’	217
	R: 5’-CGGCAGCAAGTCCAATA-3’	
GAPDH	F: 5’-AAGGCTGTGGGCAAGG-3’	238
	R: 5’-TGGAGGAGTGGGTGTCG-3’	
MMP-2	F: 5’-TTGACGGTAAGGACGGACTC-3’	153
	R: 5’-ACTTGCAGTACTCCCCATCG-3’	
MMP-9	F: 5’-TTGACAGCGACAAGAAGTGG-3’	179
	R: 5’-GCCATTCACGTCGTCCTTAT-3’	
